# Effectiveness of Virtual Reality Interventions for Adolescent Patients in Hospital Settings: Systematic Review

**DOI:** 10.2196/24967

**Published:** 2021-06-28

**Authors:** Brad Ridout, Joshua Kelson, Andrew Campbell, Kate Steinbeck

**Affiliations:** 1 Cyberpsychology Research Group Faculty of Medicine and Health The University of Sydney Sydney Australia; 2 Faculty of Business, Justice, and Behavioural Sciences Charles Sturt University Bathurst Australia; 3 Discipline of Child and Adolescent Health Faculty of Medicine and Health The University of Sydney Sydney Australia

**Keywords:** virtual reality, hospital, pain, anxiety, adolescents

## Abstract

**Background:**

Given the high level of interest and increasing familiarity with virtual reality among adolescents, there is great potential to use virtual reality to address adolescents’ unique health care delivery needs while in hospital. While there have been reviews on the use of virtual reality for specific health conditions and procedures, none to date have reviewed the full scope of virtual reality hospital interventions for adolescents who are often combined with children as a homogenous group, despite the fact that adolescents experience virtual environments different from children.

**Objective:**

The aim of this review was to systematically identify available evidence regarding the use of virtual reality interventions for adolescent patients in hospital settings to evaluate effectiveness, suitability, and safety and identify opportunities for future research.

**Methods:**

PubMed, PsycINFO, Medline, and Scopus databases were searched using keywords and phrases. Retrieved abstracts (n=1525) were double screened, yielding 276 articles for full-text screening. Of these, 8 articles met inclusion criteria. Data were extracted to a standardized coding sheet, and a narrative synthesis was performed due to the heterogeneity of the studies.

**Results:**

Four RCTs and 4 single-case reports were identified for inclusion, all of which aimed to reduce pain or anxiety. The scenarios targeted were burn pain, venipuncture, chemotherapy, preoperative anxiety, and palliative care. Three out of 4 RCTs found significant reductions in pain or anxiety outcomes measures when using virtual reality compared to standard care or other distraction techniques; however, only 1 study combined self-reported experiences of pain or anxiety with any physiological measures. Single-case reports relied primarily upon qualitative feedback, with patients reporting reduced pain or anxiety and a preference for virtual reality to no virtual reality.

**Conclusions:**

Virtual reality can provide a safe and engaging way to reduce pain and anxiety in adolescents while in hospital, particularly when virtual reality software is highly immersive and specifically designed for therapeutic purposes. As VR becomes more accessible and affordable for use in hospitals, larger and more diverse studies that capitalize on adolescents’ interest in and aptitude for virtual reality, and on the full range of capabilities of this emerging technology, are needed to build on these promising results.

**Trial Registration:**

PROSPERO International Prospective Register of Systematic Reviews CRD42020198760; https://www.crd.york.ac.uk/prospero/display_record.php?ID=CRD42020198760

## Introduction

Interest in the use of virtual reality (VR) in the health sector has increased steadily over the past decade; recent advances have made VR technology more immersive, flexible, portable, and affordable. VR has been studied in the treatment of a wide range of clinical conditions, including pain management [1-3], rehabilitation [4], anxiety [5], phobias [6], and posttraumatic stress disorder [7].

The term *VR* has been used in health research to refer to a variety of simulated experiences between an individual and a 3D computer-generated environment, including videogames using a standard monitor [8]. However, VR is now mostly considered to require immersion [9,10] in a 3D environment that provides the user with the illusory experience of being in a place other than where they physically are (known as presence) [11,12]. This is usually achieved with a stereoscopic head-mounted display, often with motion tracking that allows the user to actively determine their field of view (by moving their head) to interact with the environment.

The ability of VR to modulate subjective experience lends itself to use by patients in hospital settings, where it may be used to offer respite from stressful or confining environments, such as hospital wards or emergency departments, or as a distraction from chronic or procedural pain or anxiety [13]. While there have been reviews into particular applications of VR for specific health conditions or in-hospital procedures (eg, burn management [14], procedural pain [1]), to date there has only been 1 systematic review, which included only RCTs, on the full scope of immersive VR use in hospital settings [13]. Furthermore, reviews [1,13-15] have typically combined results for patient groups ranging from young children to older adults and have not taken into consideration differences in levels of enthusiasm, aptitude, nor predisposition toward VR between these populations [16]. Brain-imaging research has suggested that young children’s brains process virtual environments different from the manner in which adolescent and adult brains process virtual environments [17-19].

VR is particularly appealing to adolescents; a recent US survey found that 73% of adolescents aged 11 to 15 years are fairly to extremely interested in VR [20]. Adolescents, defined by the World Health Organization as aged between 10 and 19 years [21], are developmentally distinct from young children and adults in terms of neurocognitive and physical maturation [22], yet studies of the use of VR for pediatric patients to date have primarily used samples that combined adolescents with young children [23-26], which makes it difficult to determine adolescent-specific outcomes.

Hospitals may be particularly stressful environments for adolescents, who are at a vulnerable stage in their development [27]. Their health issues require different responses from the hospital system than those young children [28,29] or adults [30] require, because they are affected by the physical, emotional, psychological, and sociocultural stages of adolescence as they develop their identity and autonomy [31]. For example, adolescents with chronic health conditions have preferences and concepts of care that differ from those of adults [32,33]. Adolescents also have more awareness and knowledge of their health than younger children yet may not have the emotional or cognitive resources to deal with their situation as well as older populations [34]. Furthermore, adolescents present hospitals with unique medical and psychological challenges, such as those related to the onset of mental health disorders [35], and difficulties in ensuring compliance [36], especially when adolescents perceive their dignity to be violated [37]. It is for these reasons that a growing body of research has advocated for specialist physician training and accreditation in adolescent medicine [38,39] and for adolescents to be studied as a developmentally distinct group separate from children and adults [30].

Given the high level of interest and familiarity with VR among adolescents and VR’s increasing affordability and accessibility, there is great potential to use VR to address the unique health care delivery needs of adolescents while in hospital, both as inpatients and outpatients. It is, therefore, important to understand how VR has been used in the treatment of adolescents in hospital settings to date and whether their interest in this technology translates to its enthusiastic use for therapeutic purposes, so that researchers and physicians can leverage the potential health benefits of VR for this population.

To date, no systematic review has been conducted on the overall use of VR in hospital settings among adolescents. This systematic review is therefore needed to determine how VR is currently being used to improve the well-being and experiences of adolescents in hospital; evaluate the effectiveness, suitability, and safety of such interventions; and identify opportunities for future research.

## Methods

### Search Strategy

This systematic review was performed using PRISMA (Preferred Reporting Items for Systematic Reviews and Meta-Analyses [40]) (Multimedia Appendix 1). PubMed, PsycINFO, Medline, and Scopus databases were searched using the following search phrase: (virtual reality OR VR) AND (adolescen* OR child* OR pediatric* OR youth OR teen*) AND (hospital* OR inpatient OR treat* OR surg*). Searches were conducted in May 2020 and restricted to English-language articles published in peer-reviewed journals between January 2005 and May 2020. This review (and protocol) was registered (PROSPERO; CRD42020198760).

The database searches yielded 2214 records (549 from PubMed, 216 from PsycINFO, 592 from Medline, and 857 from Scopus), from which 689 duplicates were removed. Manual searches of previous reviews, key journals, and reference lists of key articles were conducted; however, no additional records were identified.

### Eligibility Criteria

#### Record Type

As VR research in health is still in its infancy and recent reviews of restricted populations have identified mostly exploratory and feasibility studies [41,42], empirical studies of all research designs (including single-case reports) were included, to ensure the review was comprehensive.

#### Participants

Studies with a target population of adolescents, defined as aged between 10 to 19 years [21], were included.

#### Intervention Hardware

This review was restricted to studies that used immersive forms of VR delivered through a head-mounted display. Because it is not always possible for patients using VR in health settings to move their head, this review included VR studies that were immersive but not necessarily interactive (ie, passive forms of VR such as prerecorded immersive VR videos were included). Although Cave Automatic Virtual Environment systems, which use projections to display the VR environment on walls of a special-purpose room, are considered immersive, they are far less affordable and widely used than head-mounted displays [10,43] and are seldom available in hospital settings. Studies using Cave Automatic Virtual Environment systems were, therefore, excluded, along with studies that used nonimmersive hardware such as computer monitors.

#### Intervention Settings

Studies on interventions that took place in hospital settings were included. Participants could be either inpatients or outpatients.

### Screening Process

The screening process involved 2 stages: (1) title and abstract exclusion and (2) full-text exclusion. All records were independently screened by 2 reviewers (BR and JK) in both stages to establish relevance for inclusion. Any discrepancies between reviewers’ decisions were resolved by discussion with the authors who were not involved in selection (AC and KS) until mutual agreement was reached.

### Data Extraction

Data from studies included in the review were extracted by 1 reviewer (BR) to a standardized coding sheet, which was then checked by a second reviewer (JK). Data extracted for synthesis were reference source (first author surname; year of publication), methodology (health problem or procedure under investigation; study design; risk of bias assessment), participants (country; attrition rate; sample size, age, and gender characteristics), intervention details (treatment conditions; VR equipment and software), and findings (primary outcomes, ie, impact on physical or psychological measures; usability outcomes, ie, measures of engagement, acceptability).

### Risk of Bias Assessment

Risk of bias was assessed independently by 2 reviewers (BR and JK) for RCTs, using the Cochrane Collaboration tool [44], and for single-case reports, using the Methodological Quality and Synthesis of Case Series and Case Reports Protocol [45]. Any discrepancies between reviewers were resolved by discussion. Given that it is not possible to blind participants or personnel to a VR intervention condition, the questions about performance bias and detection bias were not assessed for RCTs. An additional domain—whether any confounder variables at baseline were accounted for—was included under other risks. For case reports, causality questions (“were other alternative causes that may explain the observation ruled out?”; “was there a challenge/rechallenge phenomenon?”; “was there a dose–response effect?”) were not applicable to this study and were not assessed.

### Data Analysis

Given the small number of studies included in this review and the heterogeneity of their aims, research design, and outcome measures, quantitative meta-analysis was not possible. A narrative synthesis approach was used to describe the findings of this systematic review.

## Results

### Study Selection

The literature search yielded 2214 records. Of the 1525 records that remained after duplicate removal, 1249 were excluded because their titles and abstracts indicated that they were not about virtual reality interventions for adolescent patients in hospital settings, leaving 276 articles to be assessed for eligibility based on inclusion criteria.

**Figure 1:**
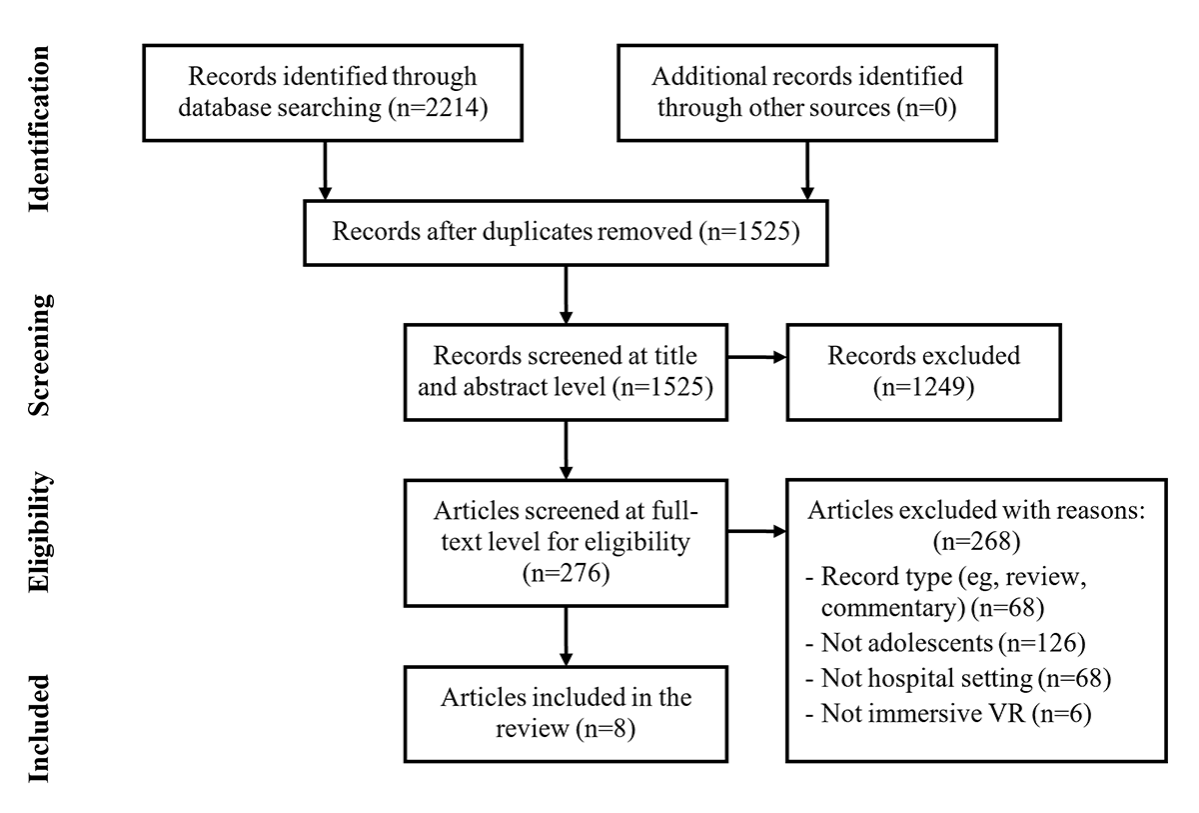
Selection flowchart.

During the second stage of screening, 68 articles were excluded based on record type; 126 were excluded because the target population was not limited to adolescents; 68 articles were excluded because the intervention did not take place in hospital settings; and 6 were excluded because they did not use immersive VR. A total of 8 studies were included (Figure 1).

### Health Problems/Procedures and Study Designs

Detailed characteristics of the included articles (n=8) are provided in Multimedia Appendix 2. Four studies [46-49] were RCTs and 4 studies [50-53] were single-case reports. Of the RCTs, 2 studies [46,49] compared VR to standard care, 1 study [47] compared VR to standard distraction (TV, music, books), and 1 study [48] compared VR to 2 control groups—standard care and passive distraction (watching a movie). The study design of 1 RCT [46] was incorrectly described as quasi-experimental and the control group was described as waitlist; however, private communication with the authors confirmed that while on the waitlist, the control group received the same standard care as the intervention group but without the VR component (G Manshaee, PhD, email communication, August 9, 2020). Given this information and the fact that group assignment was randomized, we classified the study as an RCT.

Three studies used VR for distraction from burn pain (2 RCTs [47,48], 1 case report [50]), 2 studies used VR for distraction from pain and anxiety during chemotherapy sessions (1 RCT [46], 1 case report [51]), 1 study [49] used VR for distraction from pain and anxiety during venipuncture, 1 case report used VR to reduce preoperative anxiety [52], and 1 case report used VR to improve well-being in palliative care [53].

Three of the RCTs [46,48,49] compared pretest and posttest measures of outcome variables (1 study also included 7-day and 1-month follow-ups [46]), while 1 RCT [47] measured burn pain intensity, observed pain behaviors, and objective physiological indicators (heart rate and oxygen saturation) at 3 time points: baseline, during dressing removal, and during dressing application. Two case reports [50,51] compared outcome variables measured with and without VR, while the other 2 case reports [52,53] only recorded qualitative assessments of the patients’ VR experiences.

### Risk of Bias Assessment

The risk of bias for the RCT studies (Multimedia Appendix 3) was low for most studies in most domains. Baseline confounding variables were adequately accounted for in 2 RCTs [47,49], but there was a high risk of bias for this domain in 1 RCT [46] and an unclear risk in the other RCT [48]. All of the case reports were assessed as having some risk of methodological bias (Multimedia Appendix 4). None of the case reports described clear selection methods, and 2 case reports [52,53] did not adequately ascertain outcomes (ie, results were qualitative or broadly descriptive only).

### Participant Characteristics

Studies were conducted in the United States (n=5), Australia (n=1), Iran (n=1), and Canada (n=1). The RCTs investigating VR for burn pain had similar sample sizes (28 participants [48] and 41 participants [47]) and gender distributions (approximately one-third of participants were female). In the RCT investigating VR and chemotherapy [46], all the participants were female (sample size of 30). The RCT investigating VR and venipuncture had a large sample size (143 participants) and an even gender distribution [49]. The overall attrition rate in RCTs was negligible, with 2 studies [46,47] reporting no attrition. In the other 2 studies, attrition occurred primarily due to participants withdrawing or rescheduling prior to their procedures.

The age range for all included studies fell within the World Health Organization definition of 10 to 19 years [21], with the exception of 1 RCT [49], which also included 16 participants (16/143, 11.2%) aged 20 or 21 years who were defined as adolescents in the study because they were being treated at a Children’s Hospital. The age distributions of 2 RCTs were similar (range 11-17 years, mean 13.1 years [47]; range 10-17 years, mean 13.5 years [48]), while 1 RCT [46] had an age range of 14 to 18 years (mean 14.9 years). In the case reports, 3 patients were aged between 10 and 12 years (2 males [50,52] and 1 female [53]), and 1 patient was a 17-year-old male who was morbidly obese [51].

### VR Software

Most studies tested VR programs that were specifically designed for therapeutic purposes: SnowWorld (University of Washington Harborview Burn Center and Firsthand Technology Inc), the first VR software created specifically for pain distraction during burn wound redressing [50]; Bear Blast (AppliedVR Inc), a fast-paced interactive game designed for pain distraction, for venipuncture [49]; and Ocean Journey, a passive (ie, noninteractive) VR therapy film, for chemotherapy [46]. In 1 case report [53], the patient, who was in palliative care, was provided with a range of 360° videos (Wishplay), designed to allow patients to have experiences beyond the limitations of their illness or disability, which included a figure-skating experience custom-made for the patient.

Three studies used off-the-shelf software not designed for therapeutic purposes: 1 case report on a patient undergoing chemotherapy used Ocean Descent (Sony Interactive Entertainment); 1 RCT on burn pain used age-appropriate interactive VR games (Chicken Little for 11- to 13-year-olds; Need for Speed for 14- to 17-year-olds) [47]; and 1 case report used Oculus Rooms to connect the patient suffering preoperative anxiety to their parent (who was located in the preoperative area) and allowed them to play a virtual board game together while the patient was transported to the operating table and until they lost consciousness from the anesthesia [52]. This case report was the only study included that used software that allowed virtual interaction with other users.

The VR session length in half of the studies [46,50-52] was between 20 and 30 minutes, in line with recommendations for VR session length [54]. The exceptions were the study with venipuncture [49], in which the session length was less than 5 minutes due to the short procedure; the palliative care study (5-10 minutes per video) [53], and the 2 studies with burn wound care [47,48], in which session length varied greatly depending on the length of time required to remove and apply dressings (2-100 minutes). A facilitator was present in all sessions to help guide the adolescent participants, with the exception of the palliative care study [53], in which the patient was first guided to ensure tolerability, and then left to use the VR device at their own leisure for approximately 4 weeks.

### VR Equipment

Studies used a variety of immersive VR devices (Multimedia Appendix 2). The studies with patients with burn wounds, which were the oldest studies included, each used different VR headsets connected to desktop computers with user control via joystick or trackball (ie, no head tracking): Kipping et al [47] excluded patients with burn wound locations that would impact their ability to wear a head-mounted display, while Jeffs et al [48] and Hoffman et al [50] used head-mounted displays on custom-built adjustable arm devices to allow patients with burn wounds on the head to be included. The other studies used widely available consumer head-mounted displays with head tracking capabilities (Oculus Go, Oculus Rift, Sony PlayStation VR), including 3 studies [46,49,53] that utilized smartphones as the display and processor (Samsung VR Gear, Merge VR Goggles, Google Daydream).

### Primary Measures and Outcomes

There was almost no commonality in the measures used to assess primary outcomes (Multimedia Appendix 2), with the exception of the use of a visual analog scale to assess pain in RCTs for venipuncture [49] and burn pain [47].

Gold and Mahrer [49] found that using VR for distraction during venipuncture resulted in significantly less procedural pain and procedural anxiety (each measured with visual analog scales), and significantly better affect (measured with the Facial Affect scale) for VR than that for standard care, when controlling for baseline pain. Secondary analyses revealed that patients with high anxiety sensitivity benefited the most from VR [49].

Kipping et al [47] measured burn pain intensity (with a visual analog scale) and observed pain behaviors, heart rate, and oxygen saturation, but the only significant difference between VR (using off-the-shelf interactive games) and standard distraction was fewer pain behaviors observed by nurses during dressing removal for the VR condition. Jeffs et al [48] found that procedural pain while using SnowWorld was significantly lower for VR (and was the only condition in which procedural pain was reduced compared to preprocedure pain) than that for passive distraction or standard care (adjusted for age, sex, preprocedure pain, state anxiety, opiate use, and treatment length). A patient with burns undergoing occupational therapy also reported lower pain when using SnowWorld than that experienced in standard occupational therapy sessions the days before and after the VR session [50].

Sharifpour et al [46] found that, after 8 weekly 30-minute chemotherapy sessions while watching the noninteractive VR film Ocean Journey, patients reported lower pain intensity, pain anxiety, pain catastrophizing, and pain self-efficacy compared to the standard care control group. These effects were maintained for subsequent weekly chemotherapy sessions without VR, at 7-day and 1-month follow-ups. One case report with chemotherapy [51] did not measure pain or anxiety directly; the use of VR during a monthly lumbar puncture procedure (injection of intrathecal chemotherapy) reduced the amount of analgesics and anxiolytics required by the patient by approximately half compared to the amount required in the previous 4 monthly procedures without VR, and procedure and recovery were significantly faster (42% and 30%, respectively).

Two case reports [51,52] did not use quantitative measures. In 1 study [52], staff reported that the patient remained calm and showed no signs of distress or anxiety while using VR during transportation to the operating therapy and while being administered anesthesia. In the other study [51], the patient in palliative care reported that using VR distracted them from their pain and loneliness and had a positive impact on their mood.

### Usability Measures and Outcomes

Three of the RCTs [47-49] used quantitative usability or engagement measures, but there was no commonality among them. Kipping et al [47] found self-reported presence (with a visual analog scale) while using VR to be positive but with room for improvement (mean 6.1 out of 10). Jeffs et al [48] found that engagement, using a nonstandard question about perceived ability to pay attention to the distraction (either VR or passive distraction) rated on a 5-point scale, demonstrated a significant negative correlation with both anxiety and procedural pain reduction in both groups, though a direct comparison between VR and passive distraction groups was not performed. The venipuncture RCT [49] measured usability using 2 investigator-developed Likert-type scale measures and a qualitative questionnaire: results indicated a high level of immersion and satisfaction with the VR game, and 92% of participants reported no feelings of sickness during the VR session. Jeffs et al [48] was the only other RCT to mention side-effects, with none reported. The chemotherapy RCT [46] did not report any usability or engagement data.

All case reports found the VR experiences to be both immersive and fun, with no feelings of sickness or discomfort reported; however, only 1 study (the patient in palliative care [53]) confirmed an absence of side-effects. The only usability issues that were reported were from the patient suffering preoperative anxiety (orientation disturbance) within Oculus Rooms: while the patient was being moved during transportation to the operating room (although the patient was able to quickly correct the orientation without assistance); and poor Wi-Fi connectivity [52].

## Discussion

### Principal Findings

The aim of this systematic review was to identify studies that investigated the use of immersive VR using head-mounted displays to improve the well-being and experiences of adolescents in hospital settings. We chose this age group and setting for the following reasons: First, it is often assumed that adolescents will be enthusiastic users of therapeutic digital technology because they are engaged users of similar technology for social and leisure purposes; however, there is little research evidence to date to support that this is a reasonable assumption. Second, adolescents are often viewed as challenging or hyperemotional in binary (pediatric or adult) health care settings [35,36,55]. Part of this hyperemotionality is related to neurocognitive development [56] and may be better managed with distraction or immersion using VR than by traditional models of care used for younger and older populations.

We identified 8 eligible articles (4 RCTs and 4 case reports), all of which aimed to reduce pain or anxiety. The number of articles was low, but not unremarkably so, given that research with adolescents in hospital settings is often stalled around consent and risk issues [57], and in studies, adolescents are often combined with young children or adults [57]. The health problems and procedures targeted were burn pain, venipuncture, chemotherapy, preoperative anxiety, and palliative care. While the lack of large RCTs precluded any meta-analysis, most [46,48,49] found significant reductions in pain or anxiety outcomes measures when using VR compared to those when using standard care or other distraction techniques. There was little commonality in the measures used to evaluate primary and usability outcomes, with only 1 study [47] combining self-reported pain or anxiety with physiological measures. Risk of bias was generally low for the RCTs but was relatively high for the single-case reports due to their study design and unclear selection method. Caution needs to be exercised when interpreting these findings, particularly for the case reports.

VR was well received by adolescents, who generally found it to be safe, fun, immersive, and engaging. The attrition rates in the RCT studies were very low compared to those of other VR studies [54], which supports the view that adolescents find VR more engaging than other populations, which is highly relevant.

A wide range of head-mounted displays were used, including headsets that utilized smartphones as the display and processor. Studies [48,50] also demonstrated the efficacy of using custom-mounted head-mounted displays combined with hand controllers for patients who are undergoing treatments that prevent them from moving their head. Consistent with the findings of previous studies with pediatric patients [58-61], there were very few reports of side-effects associated with VR use; however, only 1 study [49] in this review quantitatively measured feelings of nausea.

### Therapeutic Mechanisms of VR Use by Adolescents

The synthesis of findings from this review present an up-to-date account of the evidence base for VR use by adolescents in hospitals. There are sound reasons why VR might be generally an effective therapeutic intervention for pain, anxiety, and other distress associated with necessary health care situations, as well as some reasons why VR in these health care situations might be particularly advantageous for adolescents.

The mechanism by which VR reduces the experience of pain and anxiety has mostly been attributed to active distraction. That is, it directs the patient’s attention away from their treatment or condition by requiring them to interact with the VR environment. The neuromatrix theory of pain [62] suggests that the perception and experience of pain can be altered by cognitive, sensory, and affective experiences. VR interventions that actively distract by engaging cognitive resources (by being engaging and interactive), offer high sensory stimulation (by being immersive), and lead to positive affect (by being enjoyable) can therefore reduce the neurological resources available for processing pain [63,64]. Studies have shown that adolescents are more sensitive to pain and likely to become more emotionally dysregulated when faced with situations that are unfamiliar [65], so active distraction using VR may be particularly beneficial for adolescents, especially in hospital settings.

It has previously been demonstrated that active distraction using VR is particularly effective in reducing burn pain [14,66], especially when using SnowWorld, a game specifically designed for burns patients. Consistent with these findings, 2 studies [48,50] in this review that used SnowWorld reported a reduction in adolescents’ experiences of pain. In contrast, a study [47] that used off-the-shelf VR games for burn pain found that they were not significantly better than using standard distraction methods and induced only a moderate level of presence. Previous studies [67-69] have shown that the level of presence or immersion experienced by patients in VR interventions is directly correlated with the level of pain reduction. Kipping and colleagues [47] noted that any savings realized from using off-the-shelf VR software are likely to be at the expense of effectiveness.

While passive or less immersive scenarios have been shown to provide little relief from severe pain, for example, in patients with burns [70], these scenarios have been effective in chemotherapy. Sharifpour and colleagues [46] demonstrated that watching a passive VR film during weekly 30-minute chemotherapy sessions was not only effective in reducing adolescents’ scores on a range of pain and anxiety measures, but also that this effect was maintained for subsequent weekly sessions without VR. These findings suggest a mechanism other than active distraction in this study. It has been shown that VR can lead to increased cognitive control over pain, by facilitating relaxation and changing the way people think about pain [71,72], specifically by reducing pain catastrophizing, and increasing pain self-efficacy (ie, the ability to tolerate and control pain). This would fit with the concept that situations facilitating mastery and self-control are positive learning experiences for adolescents [73]. This suggests that the use of VR in the management of pain during chemotherapy, chronic pain associated with cancer, or other medical conditions may provide lasting benefits, even afterward when one is not using a VR device [74].

The patient in the palliative care case report was also experiencing chronic pain, but reported another therapeutic mechanism—immersive VR helped as a distraction from the loneliness and boredom associated with long-term hospitalization and waiting for health care attention [53]. With emerging research showing that VR use in palliative care settings is acceptable and well tolerated [75,76], more studies are needed to investigate expanding the use of VR to settings beyond distraction and relieving boredom, such as connecting adolescents with their peers and family to share a VR experience or play VR games together. Isolation from friends and peers is one of the most frequently reported negative aspects of hospitalization for adolescents [27,30]. Using VR to play games with friends or communicate with family via home-to-hospital live streaming [77] would therefore be particularly suitable for improving the mood and well-being of hospitalized adolescents, especially given their high level of enthusiasm and predisposition toward VR [16,20] and VR’s emerging acceptability for use in these settings [26].

### Implications for Future Research

The studies included in this systematic review suggest that there is tremendous potential for immersive VR to improve the well-being of adolescents in hospital settings. While the evidence base for this specific population is not yet established, the inclusion of case studies in this review demonstrates feasibility for several new applications of VR for adolescents in hospital settings and provides researchers with directions for potential interventions for the future.

This review also highlighted a number of methodological concerns that researchers in this field should seek to address in future studies. These include inconsistency across studies in the selection of primary outcome and usability measures, a lack physiological measures to complement subjective measures, and the difficulty in replicating studies due to the wide use of customized software and hardware.

Another contribution of this adolescent-specific review is the identification of several gaps in the literature for this population. The studies included in this review focused on pain and pain-related anxiety, however, recent studies in nonhospital settings have shown great potential for VR to be used to reduce other kinds of psychological distress in adolescents in a range of circumstances [78-82]. Emergency departments are a hospital environment that adolescents are more likely to use than other age groups [83], but also one that adolescents find particularly distressing due to long waits, loud sounds, bright lights, privacy intrusions, and exposure to the distress of others [84]. VR could therefore be effective in not only dealing with acute procedural pain while in emergency [15], but also in blocking out distressing stimuli for a calming experience that could assist them in regulating their emotions [79]. A recent study has demonstrated that VR use in the emergency department can significantly reduce levels of anxiety and anger in adults [85]; therefore, future studies should test whether these findings are also true in adolescents.

There is also opportunity for greater use of VR to connect adolescents in hospital with others in real time. Gupta and colleagues [52] demonstrated the feasibility of using Oculus Rooms to connect with a parent to relieve preoperative anxiety, but there are many other situations in which hospitalized adolescents are separated from their family and peers (eg, isolation rooms, palliative care, because of visiting hours limitations or having travelled from rural and remote areas). While the use of VR headsets that use smartphones as the display and processor (eg, Samsung VR Gear) is declining due to the increase in development and affordability of all-inclusive head-mounted displays (eg, Oculus Quest 2) [86-88], the use of either type of head-mounted display may be well-suited for hospitalized adolescents, who could potentially use their own smartphones to connect with others in VR, if given access to reliable Wi-Fi and the right software apps.

Future studies should seek to expand on the findings of the case reports included in this review, by empirically investigating the feasibility of using VR to connect adolescents with others to not only distract from pain and anxiety, but also improve well-being by relieving distress and boredom when hospitalized or waiting in emergency departments for extended periods. The potential for VR to improve pain self-efficacy and to better cope with chronic pain should also be explored.

### Limitations

This systematic review was limited to studies on the use of immersive VR to improve the well-being and experiences of adolescents in hospital settings. As such, studies that combined adolescents with other populations such as younger children or adults were excluded. Searches were also limited to English language publications and excluded grey literature and conference papers, as we were concerned with identifying the state of peer-reviewed research. Given the small number of studies identified, and the heterogeneity of aims, research designs, and outcome measures, it was not possible to conduct a meta-analysis.

While 8 studies may seem to be a low number for a systematic review, this is not uncommon when reviewing novel uses of emerging technology in specific settings and populations [89-92]. Given that VR devices have only recently become more affordable and portable (indicated by half of the included studies being published from 2018 onward), and hence more suitable for wide implementation in hospital settings, it is likely that more research in this area is currently in progress and has yet to be published. Alternatively, the challenges of undertaking novel research in busy hospital settings cannot be discounted as a reason for the low number of studies that were found.

The strengths of this review include a clear research question, prospectively registered protocol, thorough search strategy with more than 1 assessor, and the inclusion of all research designs to capture applications of VR at various stages of development. No prior reviews have specifically investigated the full range of immersive VR use to improve the in-hospital experiences of adolescents.

### Conclusion

This was the first systematic review of published studies on the use of immersive VR to improve the well-being and experiences of adolescents in hospital settings without confounds from younger children and adults. This is an important contribution to the field of VR health research, given that adolescents are developmentally distinct from other age groups, and present hospitals with unique challenges and health care delivery needs for which VR may be a useful and appealing tool. Studies varied in terms of quality and design, from RCTs to single-case reports that support the feasibility of potential interventions for the future. Overall, there was support for the effectiveness of VR in hospitals to reduce pain and anxiety in adolescents, particularly when VR software was highly immersive and specifically designed for therapeutic purposes.

There were examples of both active and passive distraction mechanisms being effectively used by VR interventions, although the latter is currently underutilized for adolescents in hospital settings. There was also a lack of RCTs investigating the effect of VR on adolescents in hospital without combining results with younger children, who process virtual environments differently. Future studies should use larger sample sizes and RCT designs, evaluate physiological and psychological outcome measures in addition to self-report measures, and address current gaps in the literature by empirically investigating the use of VR to relieve psychological distress in adolescents while in hospital, connect adolescents with friends and family to improve their well-being, and help adolescents develop skills to better tolerate and control chronic pain.

The use of VR in the health sector has enormous potential, especially for use with adolescents, who have a keen interest and aptitude toward this emerging treatment modality. As VR technology continues to improve and become more affordable, the evidence base for its effectiveness in relieving adolescent pain and distress in hospital settings should continue to grow.

## Appendix

Multimedia Appendix 1 PRISMA checklist.

Multimedia Appendix 2 Study characteristics.

Multimedia Appendix 3 Risk of bias - randomized controlled trials.

Multimedia Appendix 4 Risk of bias - case reports.

## Abbreviations

PRISMA: Preferred Reporting Items for Systematic Reviews and Meta-Analyses
RCT: randomized controlled trial
VR: virtual reality
